# Distinct plasma cytokine and chemokine profiles in severe COVID-19 and septic shock

**DOI:** 10.1371/journal.pone.0347126

**Published:** 2026-04-17

**Authors:** Se Ju Lee, Jaehoon Kim, Min Han, Jung Ah Lee, Yongseop Lee, Jung Ho Kim, Jin Young Ahn, Su Jin Jeong, Nam Su Ku, Joon-Sup Yeom, Jun Yong Choi

**Affiliations:** 1 Division of Infectious Diseases, Department of Internal Medicine and AIDS Research Institute, Yonsei University College of Medicine, Seoul, Republic of Korea; 2 Division of Infectious Diseases, Department of Internal Medicine, Inha University College of Medicine, Incheon, Republic of Korea; Sai Gosavi Specialty Clinic / Nano Hospitals Bangalore / Saraswati Specialty Clinic, INDIA

## Abstract

**Background:**

Severe Coronavirus Disease 2019 (COVID-19) and septic shock are both characterized by dysregulated host immune responses. While similarities and differences in immune responses between COVID-19 and bacterial sepsis have been reported, direct comparative analyses remain limited. This study aims to characterize the immunologic status of patients with COVID-19 and sepsis through plasma cytokine/chemokine analysis, thereby providing additional candidates for immunomodulatory therapy for COVID-19.

**Methods:**

We included patients diagnosed with severe COVID-19 or septic shock with lymphopenia, matched for age, sex, steroid administration, and severity. A total of 20 analytes were measured using Luminex assay.

**Results:**

A total of 36 patients were enrolled. Plasma granulocyte-macrophage colony-stimulating factor (GM-CSF) concentrations were significantly higher in the COVID-19 group (5.3 pg/ml; IQR, 3.6–16.3 vs 0.0 pg/ml; IQR, 0.0–3.6; P = 0.010). Plasma interleukin-10 (IL-10) (0.0 pg/ml; IQR, 0.0–4.8 vs 28.8 pg/ml; IQR, 7.5–51.7; P = 0.003) and IL-15 (0.0 pg/ml; IQR, 0.0–0.0 vs 0.0 pg/ml; IQR, 0.0–5.6; P = 0.024) levels were significantly higher in the sepsis group. Firth logistic regression analysis showed that plasma IL-6, IL-8, and CXCL16 levels were associated with new organ support in the sepsis group, while IL-15, CXCL16, and IL-1RA levels tended to be associated in the COVID-19 group.

**Conclusion:**

At day 7 after diagnosis, both groups exhibited active proinflammatory responses, but only the sepsis group showed prominent anti-inflammatory responses. The persistent elevation of GM-CSF in the COVID-19 group, even with steroid administration, highlights its potential as a therapeutic target and underscores the need for patient stratification in immunomodulatory trials.

## Introduction

Severe Coronavirus Disease 2019 (COVID-19) and septic shock are both characterized by dysregulated host immune responses that can lead to multiple organ failure and death [1]. The concept of “cytokine storm” has been widely used to describe the hyperinflammatory status in both diseases, but recent studies suggest that the immunopathogenesis of COVID-19 may differ from that of classical bacterial sepsis [2,3]. Several studies have reported that proinflammatory cytokine levels in COVID-19 are not as high as those observed in bacterial sepsis [2,4]. However, direct comparative studies on cytokine/chemokine profiles between severe COVID-19 and septic shock remain limited.

Understanding the similarities and differences in immune responses between COVID-19 and bacterial sepsis could provide additional candidates for immunomodulatory agents for severe COVID-19 treatment. The temporal dynamics of pro-inflammatory and anti-inflammatory mediators may differ between viral and bacterial sepsis [5]. For instance, while both diseases showed elevated levels of interleukin-6 (IL-6), IL-8, and tumor necrosis factor-alpha (TNF-α), several studies suggest that granulocyte-macrophage colony-stimulating factor (GM-CSF) may have different impacts on the treatment of COVID-19 and sepsis [5–7]. Conversely, anti-inflammatory cytokines, such as IL-10, may be more prominent in sepsis [2].

Sepsis is known to involve the simultaneous activation of proinflammatory responses and compensatory anti-inflammatory responses, ultimately leading to dysregulated host immune responses [8]. Sometimes, chronic inflammation due to persistent dysregulated immune responses results in a chronic catabolic state known as persistent inflammation, immunosuppression, and catabolism syndrome (PICS) after sepsis [9]. Patients with COVID-19 often exhibit lymphopenia, which is characteristic of the immunosuppressed state occurring after sepsis [1,8]. Lymphopenia is associated with poor prognosis in both bacterial sepsis and COVID-19, and persistent lymphopenia reflects dysregulated immune responses [1,10]. Therefore, this study aimed to characterize the immunological status of COVID-19 and sepsis patients with persistent lymphopenia, matched for age, sex, and disease severity, through plasma cytokine and chemokine analysis.

## Materials and methods

### Ethical considerations

The Institutional Review Board of the Severance Hospital, Yonsei University College of Medicine, approved this study (4-2020-0076 and 4-2016-0605), and written informed consent was obtained from all participants. The study was conducted in accordance with the Declaration of Helsinki.

### Study design

This study included patients diagnosed with severe COVID-19 or septic shock admitted to Severance Hospital, a large tertiary teaching hospital in Korea. Patients were included based on the following criteria: (1) aged 17 years or older; (2) diagnosed with either severe COVID-19 (defined as requiring oxygen therapy) or septic shock (defined as requiring vasopressors to maintain mean arterial pressure ≥65 mmHg despite adequate fluid resuscitation and having serum lactate levels ≥2 mmol/L); (3) persistent lymphopenia at 7 days after diagnosis [11]. COVID-19 was diagnosed using real-time reverse transcriptase polymerase chain reaction (PCR) tests, and the definition of sepsis followed the revised sepsis-3 definition (infection accompanied with an increase in total Sequential Organ Failure Assessment (SOFA) score ≥ 2 points). COVID-19 patients without oxygen demands and sepsis patients who did not meet the revised sepsis-3 definition were excluded [11,12]. Lymphopenia was defined as an absolute lymphocyte count less than 1.2 × 10^3^ cells/μL [13].

To compare cytokine/chemokine profiles between the study groups, each COVID-19 patient was retrospectively and manually matched with a bacterial sepsis patient based on the following characteristics: (1) age within five years; (2) sex; (3) steroid administration status, and (4) total SOFA score within four points on the index date. To compare dysregulated immune responses in COVID-19 and bacterial sepsis, plasma samples were collected on the seventh day after diagnosis from patients with persistent lymphopenia.

### Cytokine and chemokine analysis

Blood samples were collected at 7 days after diagnosis from patients admitted to Severance Hospital with severe COVID-19 (between June 1st, 2020 and July 31st, 2021) and septic shock (between March 1st, 2017 and November 30th, 2019). Plasma samples were obtained after centrifugation and stored at −80°C.

A total of 20 analytes were measured using the Luminex assay (Millipore, Darmstadt, Germany). This included IL-1β, IL-1 receptor antagonist (IL-1RA), IL-2, IL-4, IL-6, IL-7, IL-8, IL-10, IL-13, IL-15, IL-17A, FMS-like tyrosine kinase-3 ligand (Flt-3L), interferon-γ (IFN-γ), granulocyte-macrophage colony-stimulating factor (GM-CSF), and tumor necrosis factor-α (TNF-α), which were analyzed using HCYTOMAG-60K. Additionally, IL-32α, IL-35, B-cell activating factor of the TNF family (BAFF), IFN-β, and C-X-C motif chemokine ligand 16 (CXCL16) were analyzed using HCYP4MAG-64K. Dilution standards and quality controls were followed according to the manufacturer’s instructions. Samples with undetectable cytokine/chemokine concentrations were substituted with 0 for statistical analysis.

### Characteristics and laboratory data of study population

All relevant clinical and laboratory data of the patients were retrieved from the electronic medical records. Charlson Comorbidity Index was calculated at the time of admission to classify patients according to the overall comorbidity. The SOFA score was used to measure the severity of organ dysfunction. The results of laboratory tests and SOFA score were investigated based on the index date of each patient. The index date was defined as the seventh day of COVID-19 and bacterial sepsis diagnosis.

### Outcome

The primary outcome of this study was a comparison of plasma cytokine/chemokine concentrations between the two study groups. Since immune responses vary significantly with steroid administration, each group was also analyzed individually according to this parameter. Secondary outcomes included in-hospital mortality, length of hospital stay, and new organ support. New organ support was defined as the application of invasive mechanical ventilation or continuous renal replacement therapy during treatment.

### Statistical analysis

Differences in patient characteristics and outcomes between the two groups were assessed using Chi-square test or Fisher’s exact test for categorical variables, and Wilcoxon rank-sum test for continuous variables. Firth’s logistic regression analysis was performed to assess factors associated with new organ support during treatment. Given the small sample size and limited number of events, only univariate Firth logistic regression analysis was performed to minimize model overfitting and unstable estimates [14]. Statistical significance was set at P < .05. All statistical analyses were performed using R V.4.0.5 software (The R Foundation for Statistical Computing, Vienna, Austria).

## Results

### Characteristics of the study population

This study enrolled 18 patients diagnosed with COVID-19 and 18 patients diagnosed with sepsis (Table 1). No significant differences were observed between the two groups in age [median 75.5 years; Interquartile range (IQR), 64.0–78.0 and 74 years; IQR, 65.0–83.0; P = 0.862], sex, proportion of steroid administration, lymphocyte count (0.8 × 10^3^/μL; IQR 0.5–1.0 × 10^3^ and 0.8 × 10^3^/μL; IQR 0.5–1 × 10^3^; P = 0.874), and SOFA score (2.0; IQR 1.0–4.0 and 2.5; IQR 2.0–7.0; P = 0.573). The Charlson comorbidity index also showed no significant difference between the two groups. Platelet count (215.5 × 10^3^/μL; IQR 153.0–336.0 × 10^3^ and 115.5 × 10^3^/μL; IQR 69.0–229.0 × 10^3^, P = 0.048) and serum albumin (3.2 mg/dL; IQR, 3.0–3.5 and 2.4 mg/dL; IQR, 2.2–2.6; P < 0.001) were significantly lower in the sepsis group. D-dimer (600.5 ng/mL; IQR, 204.0–1908.0 and 3094.0 ng/mL; IQR, 1278.0–9780.5; P = 0.006), C-reactive protein (16.2 mg/L; IQR, 5.9–33.5 and 45.3 mg/L; IQR, 25.1–75.7; P = 0.011), procalcitonin (0.1 ng/mL; IQR, 0.0–0.3 and 27.9 ng/mL; IQR, 3.2–72.4; P < 0.001), and arterial lactate (1.1 mmol/L; IQR, 0.8–1.3 and 1.3 mmol/L; IQR, 1.2–1.7; P = 0.048) were significantly higher in the sepsis group. There were no significant differences between the two groups in terms of in-hospital mortality, new organ support, and in-hospital stay days. Clinical trajectories from day of admission to the 7^th^ day after diagnosis are summarized in S1 Table in S1 File.

**Table 1:** Characteristics and outcomes of the study subjects.

	COVID-19 (n = 18)	Sepsis (n = 18)	P-value
Age, median (IQR), y	75.5 (64.0–78.0)	74.0 (65.0–83.0)	0.862
Male Sex, n (%)	9 (50.0%)	9 (50.0%)	>0.99
Comorbidities, n (%)			
Hypertension	12 (66.7%)	11 (61.1%)	>0.99
Heart failure	0	1 (5.6%)	>0.99
Diabetes mellitus	7 (38.9%)	7 (38.9%)	>0.99
Underlying lung disease	1 (5.6%)	3 (16.7%)	0.596
Chronic kidney disease	5 (27.8%)	2 (11.1%)	0.400
Chronic liver disease	3 (16.7%)	3 (16.7%)	>0.99
Solid cancer	4 (22.2%)	6 (33.3%)	0.710
Hematologic malignancy	1 (5.6%)	1 (5.6%)	>0.99
Cerebrovascular accident	5 (27.8%)	3 (16.7%)	0.688
Charlson comorbidity index, median (IQR)	5.0 (3.0–6.0)	5.0 (4.0–6.0)	0.949
Steroid administration, n (%)	7 (38.9%)	7 (38.9%)	>0.99
Laboratory test, median (IQR)			
White blood cell, 10^3^/μL	6.5 (4.0–8.8)	8.3 (4.3–11.7)	0.279
Lymphocyte count, 10^3^/μL	0.8 (0.5–1.0)	0.8 (0.5–1.0)	0.874
Platelet count, 10^3^/μL	215.5 (153.0–336.0)	115.5 (69.0–229.0)	**0.048**
Aspartate aminotransferase, IU/L	26.5 (22.0–37.0)	24.0 (17.0–40.0)	0.516
Alanine aminotransferase, IU/L	25.0 (19.0–43.0)	23.0 (19.0–36.0)	0.601
Total bilirubin, mg/dL	0.5 (0.3–0.7)	0.7 (0.5–1.0)	0.064
Albumin, mg/dL	3.2 (3.0–3.5)	2.4 (2.2–2.6)	**<0.001**
Creatinine, mg/dL	0.7 (0.5–1.3)	0.8 (0.6–0.9)	0.849
Ferritin, ng/mL	314.4 (188.0–770.5)	485.6 (202.0–777.0)	0.737
D-dimer, ng/mL	600.5 (204.0–1908.0)	3094.0 (1278.0–9780.5)	**0.006**
C-reactive protein, mg/L	16.2 (5.9–33.5)	45.3 (25.1–75.7)	**0.011**
Procalcitonin, ng/mL	0.1 (0.0–0.3)	27.9 (3.2–72.4)	**<0.001**
Arterial lactate, mmol/L	1.1 (0.8–1.3)	1.3 (1.2–1.7)	**0.048**
SOFA, median (IQR)	2.0 (1.0–4.0)	2.5 (2.0–7.0)	0.573
Outcome			
In-hospital mortality, n (%)	1 (5.6%)	2 (11.1%)	>0.99
In-hospital stay days, median (IQR), d	18.0 (13.0–22.0)	18.0 (12.0–26.0)	0.590
New organ support, n (%)	3 (16.7%)	4 (22.2%)	>0.99

Abbreviations: *IQR*, Interquartile range; *SOFA*, Sequential Organ Failure Assessment.

### Comparison of cytokine/chemokine profiles between the study groups

Table 2 presents the comparison of plasma cytokine/chemokine concentrations between the study groups. Plasma GM-CSF concentrations were significantly higher in COVID-19 patients (5.3 pg/ml; IQR, 3.6–16.3 and 0.0 pg/ml; IQR, 0.0–3.6; P = 0.010). Plasma IL-10 (0.0 pg/ml; IQR, 0.0–4.8 and 28.8 pg/ml; IQR, 7.5–51.7; P = 0.003) and IL-15 (0.0 pg/ml; IQR, 0.0–0.0 and 0.0 pg/ml; IQR, 0.0–5.6; P = 0.024) levels were significantly higher in sepsis patients. Plasma TNF-a (19.4 pg/ml; IQR, 13.7–27.6 and 34.2 pg/ml; IQR, 20.4–55.1; P = 0.064) and IL-17A (0.0 pg/ml; IQR, 0.0–0.0 and 5.3 pg/ml; IQR, 0.0–10.5; P = 0.069) levels were relatively lower in COVID-19 patients. Other cytokines, including IL-1β, IL-4, IL-7, and IL-13, showed no significant plasma concentrations and were mostly undetectable. Detailed information on plasma cytokine/chemokine is provided in S2 Table in S1 File.

**Table 2:** Cytokine and chemokine concentrations of the study subjects.

	COVID-19 (n = 18)	Sepsis (n = 18)	P-value
Median (IQR), pg/ml^a^			
GM-CSF	5.3 (3.6–16.3)	0.0 (0.0–3.6)	**0.010**
TNF-α	19.4 (13.7–27.6)	34.2 (20.4–55.1)	0.064
IL-1RA	51.7 (32.2–92.0)	62.2 (39.2–81.9)	0.743
IL-6	0.0 (0.0–14.3)	11.4 (0.0–31.9)	0.137
IL-8	3.1 (0.0–26.8)	10.2 (0.0–33.7)	0.487
IL-10	0.0 (0.0–4.8)	28.8 (7.5–51.7)	**0.003**
IL-15	0.0 (0.0–0.0)	0.0 (0.0–5.6)	**0.024**
IL-17A	0.0 (0.0–0.0)	5.3 (0.0–10.5)	0.069
CXCL16	980.5 (835.2–1068.7)	1035.3 (896.1–1281.3)	0.214

Abbreviations: *GM-CSF*, granulocyte macrophage colony-stimulating factor; *TNF-α*, tumor-necrosis factor-α; *IL-1RA*, Interleukin-1 receptor antagonist; *CXCL16*, C-X-C motif chemokine ligand 16.

^a^Samples with undetectable cytokine/chemokine concentrations were regarded as 0 pg/ml.

### Association of cytokine/chemokine level with prognosis

Firth logistic regression analysis showed that plasma IL-6 [odds ratio (OR), 1.13; confidence interval (CI), 1.02–1.41; P = 0.003], IL-8 (OR, 1.10; CI, 1.02–1.26; P = 0.005), CXCL16 (OR, 1.00; CI, 1.00–1.01; P = 0.015) level were associated with new organ support in the sepsis group (Table 3). In the COVID-19 group, plasma IL-15 (OR, 1.25; CI, 0.98–1.86; P = 0.071), CXCL16 (OR, 1.00; CI, 1.00–1.01; P = 0.068), and IL-1RA (OR, 1.02; CI, 1.00–1.04; P = 0.064) level tended to be associated with new organ support.

**Table 3:** Firth logistic regression analysis for new organ support in patients with sepsis and COVID-19.

	COVID-19			Sepsis		
	OR	95% CI	P-value^a^	OR	95% CI	P-value^a^
GM-CSF	0.98	0.81–1.09	0.708	0.99	0.79–1.11	0.863
TNF-α	1.01	0.98–1.04	0.313	1.01	0.99–1.04	0.174
IFN-γ	1.00	0.49–1.05	0.986	0.96	0.73–1.08	0.566
IL-1RA	1.02	1.00–1.04	0.064	1.00	1.00–1.01	0.471
IL-6	1.02	0.98–1.05	0.296	1.13	1.02–1.41	**0.003**
IL-8	1.02	0.97–1.07	0.465	1.10	1.02–1.26	**0.005**
IL-10	1.03	0.99–1.06	0.137	1.01	0.99–1.04	0.363
IL-15	1.25	0.98–1.86	0.071	1.05	0.88–1.22	0.549
IL-17A	1.00	0.90–1.04	0.891	0.99	0.84–1.12	0.900
BAFF	1.56	0.61–3.83	0.329	0.94	0.50–1.36	0.769
CXCL16	1.00	1.00–1.01	0.068	1.00	1.00–1.01	**0.015**
WBC < 4000/μL	0.27	0.00–3.64	0.363	0.26	0.00–3.36	0.338
Lymphocyte <500/μL	0.77	0.01–12.97	0.873	2.14	0.16–22.84	0.532
Steroid administration	3.18	0.34–41.79	0.309	1.73	0.21–14.58	0.600

Abbreviations: *OR*, Odds ratio; *CI*, Confidence interval; *GM-CSF*, granulocyte macrophage colony-stimulating factor; *TNF-α*, tumor-necrosis factor-α; *IFN-*γ, interferon-γ; *IL-1RA*, Interleukin-1 receptor antagonist; *BAFF*, B-cell-activating factor of the TNF family; *CXCL16*, C-X-C motif chemokine ligand 16; *WBC*, White blood cell count.

^a^P-value is calculated by Wald test for univariate Firth logistic regression.

### Comparison of cytokine/chemokine profiles between the study groups according to steroid administration status

In patients who received steroids, plasma GM-CSF concentrations remained significantly higher in the COVID-19 group (16.3 pg/ml; IQR, 9.8–20.0 and 0.0 pg/ml; IQR, 0.0–3.9; P = 0.019). Plasma IL-15 (0.0 pg/ml; IQR, 0.0–0.0 and 4.444 pg/ml; IQR, 0.0–5.120; P = 0.031), and IL-17A (0.0 pg/ml; IQR, 0.0–0.0 and 9.922 pg/ml; IQR, 0.0–12.937; P = 0.031) were lower in the COVID-19 group. Plasma TNF-a (18.8 pg/ml; IQR, 12.2–20.9 and 35.7 pg/ml; IQR, 21.2–71.3; P = 0.073) and IL-10 (0.0 pg/ml; IQR, 0.0–7.8 and 28.4 pg/ml; IQR, 5.8–47.7; P = 0.076) were also relatively lower in COVID-19 patients who received steroid treatment (S1 Fig in S1 File).

In patients who did not receive steroids, plasma IL-10 (0.0 pg/ml; IQR, 0.0–2.4 and 29.1 pg/ml; IQR, 9.6–51.3; P = 0.017) and BAFF (0.0 pg/ml; IQR, 0.0–0.0 and 1.8 pg/ml; IQR, 0.0–2.5; P = 0.032) were significantly lower in the COVID-19 group. Plasma IL-2 levels were also relatively lower in the COVID-19 group, though not statistically significant (0.0 pg/ml; IQR, 0.0–0.0 and 0.0 pg/ml; IQR, 0.0–1.734; P = 0.078). Plasma TNF-α, IL-6, and IL-8 levels showed no significant differences between the two groups (S2 Fig in S1 File).

## Discussion

This study analyzed plasma cytokine/chemokine concentrations in patients with severe COVID-19 and septic shock. Plasma cytokine/chemokine profiles were significantly different between the study groups. COVID-19 patients had significantly higher GM-CSF levels and lower IL-10 and IL-15 levels than septic shock patients, highlighting GM-CSF as a potential therapeutic target for COVID-19, even in patients receiving steroid treatment.

GM-CSF is a proinflammatory cytokine whose production is induced by various cells upon immune stimulation, such as bacterial toxins or other inflammatory cytokines [15]. High GM-CSF concentrations have been detected in bronchoalveolar lavage fluid from patients with acute respiratory distress syndrome (ARDS), which was associated with poor prognosis [15]. Several studies also showed high GM-CSF expression in CD4 T cells from patients with COVID-19 and sepsis [16,17]. Given the pathological role of GM-CSF in COVID-19, several studies have evaluated the efficacy of GM-CSF antagonists—which were previously trialed for conditions like rheumatoid arthritis and spondyloarthropathy—with promising results [18,19]. In this study, GM-CSF concentrations were higher in the COVID-19 group compared to the sepsis group, which may indicate a more prominent role for GM-CSF in the inflammatory response of COVID-19 than in bacterial sepsis. Furthermore, GM-CSF concentrations remained high in the COVID-19 group, even among patients receiving steroids. The high GM-CSF levels even after steroid administration provide a theoretical background that anti-GM-CSF therapy could be effective as an adjunctive therapy to dexamethasone, which is standard care for COVID-19 patients with oxygen requirements [12]. However, clinical trials of anti-GM-CSF therapy have not consistently demonstrated efficacy across all COVID-19 patients, suggesting that identifying specific patient subgroups most likely to benefit is crucial for future therapeutic trials.

IL-10 is generally known as an immunosuppressive cytokine, suppressing the production of pro-inflammatory cytokines such as TNF-α, IL-1, IL-6, IFN-γ, and GM-CSF [20]. IL-10 is also known to be associated with persistent immunosuppression after sepsis [9]. In this study, plasma IL-10 concentrations were lower in the COVID-19 group, showing a significant difference from the sepsis group. Low IL-10 levels compared to proinflammatory cytokines suggest that the immunosuppressive response in the COVID-19 group at 7 days after diagnosis is less prominent than in the sepsis group. Previous studies reported higher late IL-10 concentrations in severe COVID-19 than in mild or moderate cases, and high IL-10 levels were associated with poor prognosis in COVID-19 [21,22]. However, IL-10 concentrations were not significantly associated with poor prognosis in this study. The discrepancy between previous IL-10 findings and this study’s results may stem from differences in steroid administration, as previous studies showed steroid administration induced IL-10 elevation [23].

IL-15 inhibits sepsis-induced apoptosis of natural killer (NK) cells, dendritic cells, and CD8 T cells, thereby contributing to the recruitment of neutrophils at the inflammatory sites [24,25]. Accordingly, the efficacy of recombinant IL-15 therapy in sepsis has been suggested in experiments using animal models [24]. However, Abers et al. reported that increased IL-15 concentrations in COVID-19 patients were associated with poor prognosis [25]. In this study, high IL-15 concentrations also tended to be associated with poor prognosis in the COVID-19 group. Given that IL-15 improves both humoral and cellular responses, high IL-15 levels may reflect a hyperinflammatory state, leading to worsening conditions in COVID-19 patients. Nevertheless, the high IL-15 concentrations in the sepsis group in this study suggest differences in proinflammatory responses between COVID-19 and bacterial sepsis.

The anti-inflammatory cytokine IL-1RA binds to the IL-1 receptor, inhibiting innate immune response mediated by IL-1 [26]. Several studies have reported that high IL-1RA is associated with severity and poor prognosis in COVID-19 [22,27]. Thus, high IL-1RA is considered to reflect the hyperactive immune response in COVID-19. In this context, anakinra, a recombinant human IL-1RA, has been investigated for its potential role in COVID-19 treatment and has shown promising results [28]. Consistent with previous studies, high IL-1RA also tended to be associated with poor prognosis in COVID-19 in this study.

IL-6 is a potent proinflammatory cytokine and is recognized as an early biomarker and prognostic factor in sepsis [29]. Previous studies reported that persistent elevation of IL-6 was associated with long-term mortality [30,31]. Similarly, this study also showed that high IL-6 concentrations at 7 days after sepsis diagnosis might be associated with poor prognosis. However, in this study, IL-6 levels at 7 days after diagnosis were not clearly associated with poor prognosis in COVID-19 patients. Although IL-6 antagonists such as tocilizumab are currently used to treat severe COVID-19, some reports suggest that early administration of tocilizumab is more effective, so our findings may reflect the temporal changes in IL-6 levels [12,32].

IL-8 is produced by many cell types, including monocytes, lymphocytes, and granulocytes, and is released under inflammatory conditions to recruit neutrophils to inflammatory sites [33]. Previous studies reported that IL-8 concentrations at sepsis admission were associated with poor outcomes [34,35]. Furthermore, a study by Kraft et al. reporting that persistent elevation of IL-8 was associated with multiple organ failure is consistent with our findings [36].

Soluble CXCL16 acts as a chemotactic gradient for leukocytes expressing CXCR6 [37]. Several studies support the role of soluble CXCL16 as a biomarker of inflammation and its association with poor prognosis in sepsis and COVID-19 [27,37–39]. Consistent with previous studies, CXCL16 concentrations at 7 days after diagnosis of sepsis and COVID-19 showed possible associations with poor prognosis in this study.

Collectively, both COVID-19 and sepsis exhibited activated proinflammatory responses at 7 days after diagnosis. However, while anti-inflammatory responses, represented by IL-10, were also activated in sepsis, these responses were not prominent in COVID-19. Our results suggest that the immune response in COVID-19 is not merely a state of excessive inflammation, but rather a profound failure of regulatory mechanisms to balance the inflammatory surge, as suggested by previous studies [40]. Furthermore, the observation that GM-CSF remains significantly elevated in COVID-19 even under steroid treatment underscores its role as a key pathological driver that bypasses conventional anti-inflammatory therapy. The findings of this study deepen the understanding of immune responses in viral and bacterial sepsis and support the need for precision immunotherapies.

This study has several limitations. First, the small number of study subjects may limit the generalizability of this study. For COVID-19 treatment, steroids are considered standard care [12]. However, steroids for sepsis management are recommended in more limited situations, restricting the number of matched study participants. Nevertheless, the matched design and rigorous methodology support the robustness of the study results despite the small sample size. Second, the lack of lymphocyte subtypes or cell-mediated immunity measurements is also a limitation of this study. However, this study is meaningful in that plasma cytokines/chemokines reflect aspects of the immune system as effector molecules. Third, during the study period, the Delta and Alpha variants were the predominant VOCs of SARS-CoV-2 in Korea [41]. Thus, our findings are based on different SARS-CoV-2 variants compared to recent variants. However, since cytokine production does not show significant differences based on variants, the results of this study are considered valid regardless of SARS-CoV-2 variants [42]. Fourth, as cytokines and chemokines were measured at a single time point, we could not evaluate the longitudinal dynamics of the immune response over time. Fifth, the primary sources of infection in the sepsis group in our study were heterogeneous. However, systemic host response is strongly influenced by disease severity than the etiology of sepsis [43]. Given that the sepsis group in our study was consistently composed with septic shock, we consider our analysis remains valid despite the varied source of infection. Sixth, several cytokines and chemokines were measured at lower or below the detectable range than expected. These results may have been influenced by therapeutic interventions, such as corticosteroid administration, as well as pre-analytical factors, including potential analyte degradation during transport or storage.

In conclusion, this study demonstrates distinct immunological features between matched severe COVID-19 and septic shock patients. At 7 days after diagnosis, proinflammatory responses were clearly evident in both COVID-19 and sepsis. However, unlike sepsis, anti-inflammatory responses were not prominent in COVID-19. The persistent elevation of GM-CSF in the COVID-19 group, even with steroid administration, highlights its potential as a therapeutic target and underscores the need for patient stratification in immunomodulatory trials.

## Supporting information

S1 File S1 Table. Clinical trajectories from admission to day 7 after diagnosis. **S2 Table.** Additional cytokine and chemokine profiles of the study subjects. **S1 Fig.** Comparison of cytokine/chemokine profiles between steroid administration groups. **S2 Fig.** Comparison of cytokine/chemokine profiles between non-steroid administration groups.
(ZIP)
