# Supplementary material for: Distinct plasma cytokine and chemokine profiles in severe COVID-19 and septic shock
Source: PLoS One. 2026 Apr 17;21(4):e0347126. doi: 10.1371/journal.pone.0347126 (PMC13089746; doi:10.1371/journal.pone.0347126)
Supplement: S1 File — Clinical trajectories from admission to day 7 after diagnosis. S2 Table. Additional cytokine and chemokine profiles of the study subjects. S1 Fig. Comparison of cytokine/chemokine profiles between steroid administration groups. S2 Fig. Comparison of cytokine/chemokine profiles between non-steroid administration groups. (ZIP) [file pone.0347126.s001.zip › S2 Table.docx]

**S2 Table. Additional cytokine and chemokine profiles of the study subjects**

|  | **COVID-19 (n=18)** | **Sepsis (n=18)** | **P-value** |
| --- | --- | --- | --- |
| Median (IQR), pg/ml^a^ |  |  |  |
| IFN-β | 0.0 (0.0–0.0) | 0.0 (0.0–0.0) | 0.188 |
| IFN-γ | 0.0 (0.0–5.0) | 0.0 (0.0–6.6) | 0.806 |
| IL-1β | 0.0 (0.0–0.0) | 0.0 (0.0–0.0) | 0.180 |
| IL-2 | 0.0 (0.0–0.0) | 0.0 (0.0–0.0) | 0.075 |
| IL-4 | 0.0 (0.0–0.0) | 0.0 (0.0–0.0) | 0.331 |
| IL-7 | 0.0 (0.0–0.0) | 0.0 (0.0–0.0) | 0.345 |
| IL-13 | 0.0 (0.0–0.0) | 0.0 (0.0–0.0) | 0.133 |
| IL-32α | 0.0 (0.0–0.0) | 0.0 (0.0–0.0) | 0.198 |
| IL-35 | 0.0 (0.0–0.0) | 0.0 (0.0–0.0) | 0.182 |
| BAFF | 0.0 (0.0–0.0) | 0.0 (0.0–2.4) | 0.161 |
| Flt-3L | 0.0 (0.0–0.0) | 0.0 (0.0–0.0) | 0.333 |

Abbreviations: *IQR*, Interquartile range; *IFN-β*, interferon-β; *IL-2*, Interleukin-2; *BAFF*, B-cell-activating factor of the TNF family; *Flt-3L*, FMS-like tyrosine kinase-3 ligand.

^a^Samples with non-detectable cytokine/chemokine concentrations were considered to be zero pg/ml.
