# Supplementary material for: Distinct plasma cytokine and chemokine profiles in severe COVID-19 and septic shock
Source: PLoS One. 2026 Apr 17;21(4):e0347126. doi: 10.1371/journal.pone.0347126 (PMC13089746; doi:10.1371/journal.pone.0347126)
Supplement: S1 File — Clinical trajectories from admission to day 7 after diagnosis. S2 Table. Additional cytokine and chemokine profiles of the study subjects. S1 Fig. Comparison of cytokine/chemokine profiles between steroid administration groups. S2 Fig. Comparison of cytokine/chemokine profiles between non-steroid administration groups. (ZIP) [file pone.0347126.s001.zip › S1 Table.docx]

**S1 Table. Clinical trajectories from admission to day 7 after diagnosis**

|  | **COVID-19 (n=18)** | **Sepsis (n=18)** | **P-value** |
| --- | --- | --- | --- |
| Steroid Therapy |  |  |  |
| Total patients treated, n (%) | 7 (38.9%) | 7 (38.9%) |  |
| Steroid type at Day 7 | Dexamethasone (n=7) | Hydrocortisone (n=7) |  |
| Dose at Day 7, median (IQR) | 6 mg (4–6) | 50 mg (50–75) |  |
| Vasopressor (Norepinephrine) apply |  |  |  |
| At admission, n (%) | 1 (5.6%) | 18 (100.0%) | <0.001 |
| At sampling (Day 7), n (%) | 2 (11.1%) | 4 (22.2%) | 0.655 |
| Ventilator support |  |  |  |
| At admission, n (%) | 3 (16.7%) | 1 (5.6%) | 0.596 |
| At sampling (Day 7), n (%) | 2 (11.1%) | 1 (5.6%) | >0.99 |
| Continuous renal replacement therapy |  |  |  |
| At admission, n (%) | 1 (5.6%) | 4 (22.2%) | 0.335 |
| At sampling (Day 7), n (%) | 1 (5.6%) | 3 (16.7%) | 0.596 |
| Abbreviations: *IQR*, Interquartile range | | | |
