# Supplementary figures and images for: Distinct plasma cytokine and chemokine profiles in severe COVID-19 and septic shock
Source: PLoS One. 2026 Apr 17;21(4):e0347126. doi: 10.1371/journal.pone.0347126 (PMC13089746; doi:10.1371/journal.pone.0347126)

**S2 Fig. Comparison of cytokine/chemokine profiles between non-steroid administration groups**

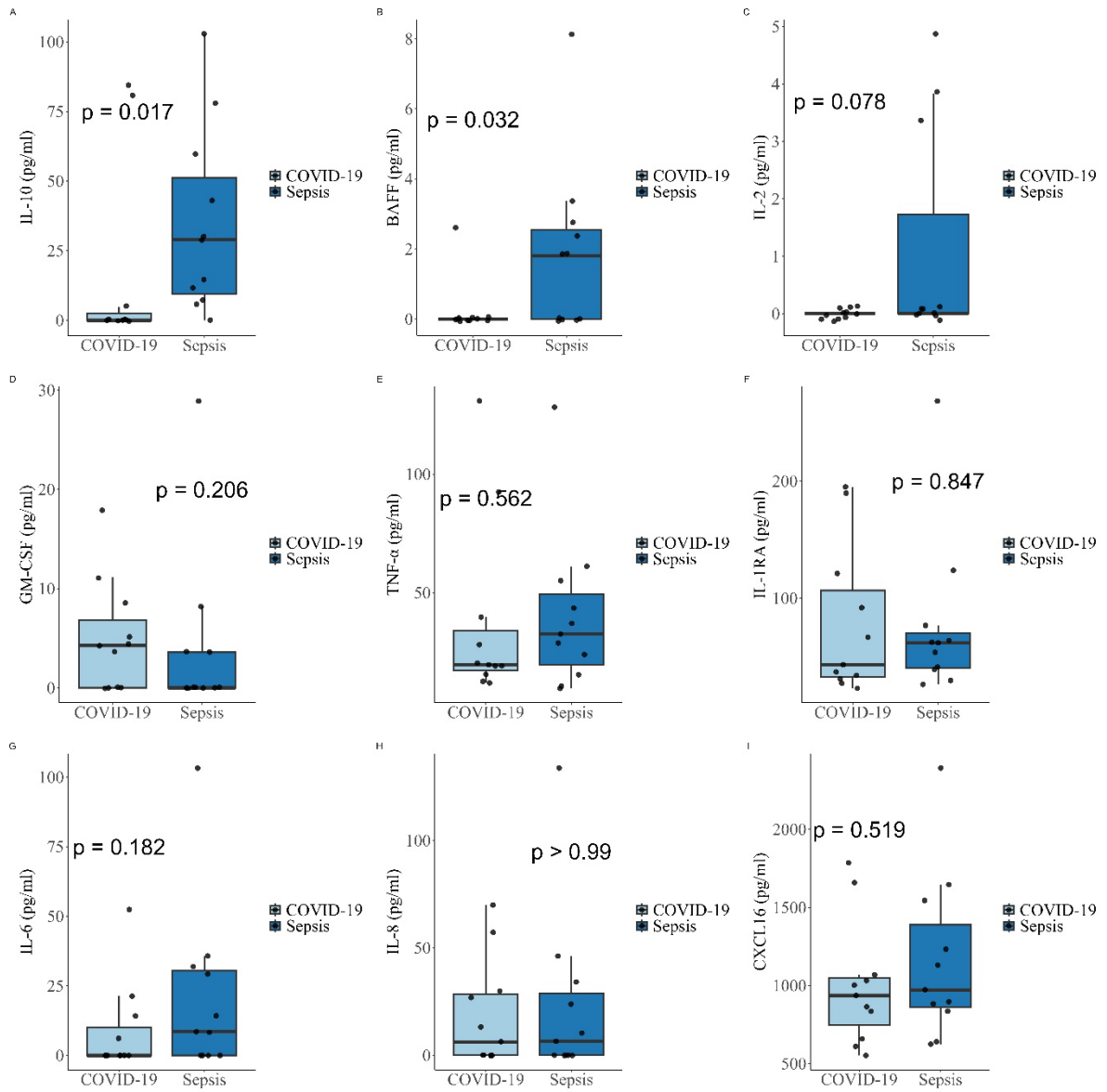

Supplement: S1 File — Clinical trajectories from admission to day 7 after diagnosis. S2 Table. Additional cytokine and chemokine profiles of the study subjects. S1 Fig. Comparison of cytokine/chemokine profiles between steroid administration groups. S2 Fig. Comparison of cytokine/chemokine profiles between non-steroid administration groups. (ZIP) [file pone.0347126.s001.zip › S2_Fig.pdf]

**S1 Fig. Comparison of cytokine/chemokine profiles between steroid administration groups**

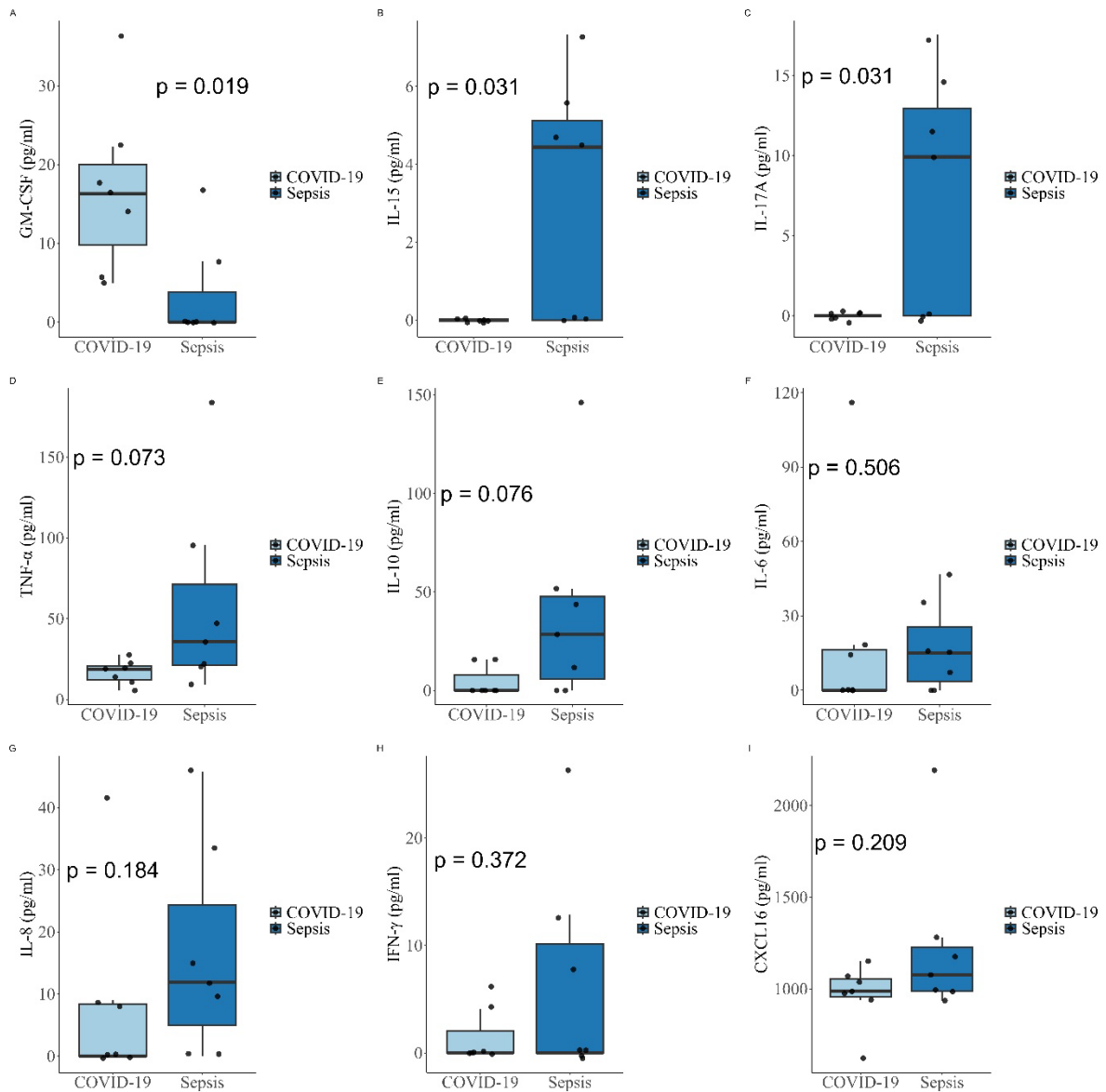

Supplement: S1 File — Clinical trajectories from admission to day 7 after diagnosis. S2 Table. Additional cytokine and chemokine profiles of the study subjects. S1 Fig. Comparison of cytokine/chemokine profiles between steroid administration groups. S2 Fig. Comparison of cytokine/chemokine profiles between non-steroid administration groups. (ZIP) [file pone.0347126.s001.zip › S1_Fig.pdf]
